# Knowledge and practice of cattle handlers on antibiotic residues in meat and milk in Kwara State, Northcentral Nigeria

**DOI:** 10.1371/journal.pone.0257249

**Published:** 2021-10-14

**Authors:** Mary Idowu Olasoju, Taiwo Israel Olasoju, Oluwawemimo Oluseun Adebowale, Victoria Olusola Adetunji

**Affiliations:** 1 Department of Veterinary Public Health and Preventive Medicine, College of Veterinary Medicine, Federal University of Agriculture, Abeokuta, Ogun State, Nigeria; 2 Department of Veterinary Public Health and Preventive Medicine, Faculty of Veterinary Medicine, University of Ibadan, Ibadan, Oyo State, Nigeria; 3 Department of Veterinary and Pest Control Services, Federal Ministry of Agriculture and Rural Development, Abuja, FCT, Nigeria; Beni Suef University Faculty of Veterinary Medicine, EGYPT

## Abstract

**Objectives:**

Antibiotics are important for improving animal health and production. However, the deposition of its residues in food of animal origin intended for human consumption at non-permissible levels has generated global health concern and the need to tackle this using the “One Health Approach”. This study assessed the knowledge and practice of 286 cattle handlers in Kwara State, Nigeria.

**Methods:**

A web-based cross sectional online survey using a semi-structured questionnaire was conducted from November to December, 2019. Univariate, bivariate and multivariate analyses were performed at 95% confidence interval to determine predictors of good knowledge and practices towards Antibiotic Residues in Meat and Milk among cattle handlers.

**Results:**

This study revealed that majority (52.7% n = 165/286) of the cattle handlers were not aware of antibiotic residues. Knowledge and practices regarding antibiotic residues were generally poor among the study population; 36.7% and 35.5% had satisfactory knowledge and practice respectively. The age (*p* = 0.026), gender (*p* = 0.006) and business duration (*p* = 0.001) of participants were significantly associated with their knowledge of antimicrobial residues. The effect of education on knowledge was modified by age. The odds of having poor knowledge on antibiotic residues increased 4 times among participants who were ≤40 years old than those above 40 years (Stratum Specific OR = 3.65; CI = 1.2, 11.1; *p* = 0.026). Knowledge levels of participants were statistically associated with their practice levels p<0.05 (OR = 2.43; CI = 1.45. 4.06; *p* = 0.0006).

**Conclusion:**

This implies that poor knowledge is a risk factor to having poor practice among cattle handlers. Deliberate efforts towards educating cattle farmers on best farm practices in antibiotic use would prevent antibiotic residues in meat and milk. Also, an effective surveillance system for monitoring the use of veterinary drugs in Kwara State, Nigeria is crucial.

## Introduction

Nigeria is one of the four leading livestock producers in Sub-Sahara Africa. The livestock sub-sector is an important and integral component of Nigeria’s agriculture and a major source of food security. Cattle are the single most important livestock species in terms of outputs and capital value [1]. In 2007, Nigeria’s national livestock population was estimated to consist of 16 million cattle [2, 3].

Antimicrobial drugs in animals are used for three major purposes namely: therapeutic, prophylactic and as growth promoters [4, 5]. Previous studies have confirmed the inappropriate use of these drugs in animals by livestock owners and pastoralists [6]. The indiscriminate use of antibiotics in animals has been linked to accumulation of antibiotic residues in foods of animal sources intended for consumption and selection pressure for antibiotic resistant bacteria in both animals and humans.

The excessive use of antimicrobial drugs in animals results in deposition of residues in meat, milk and eggs, which eventually leads to bioaccumulation of these residues in humans include carcinogenicity, mutagenicity, teratogenicity, nephropathy, hepatotoxicity, bone marrow toxicity and allergy [7].

The presence of these residues in foods of animal origin has become a major problem globally over the years, most especially in the low and middle income countries (LMICs) including Nigeria [8]. Meat and milk are highly consumed food items in the world which have also a great value for human health. Indeed, the importance of meat and milk of animals as essential sources of protein cannot be disputed; similarly, the danger and effects of antibiotic residues and resistance cannot be ignored [9].

It has been estimated that by the year 2050, antimicrobial resistance will be causing 10 million deaths annually worldwide and this will cost the world 100 trillion dollars. If left unattended to, this crisis will have worse effect as compared to the Human Immuno-Deficiency Virus (HIV) and Tuberculosis (TB) pandemic [10]. Interventions to reduce the burden of AMR have been launched worldwide due to the facts that public and economic burden has increased almost exponentially. Antibiotic residues above the International recommended permissible levels are prevalent in Nigeria and there is an urgent need for a coordinated national response to AMR [11].

While it is conceivable that resistant organisms in domestic animals could have been acquired from human and other sources, the high levels of antimicrobial residues in meat and milk point to antimicrobial use in agricultural and veterinary practices as the principal driver of resistance in Nigeria [11].

Furthermore, knowledge, belief, perception, expectations, and attitudes of people towards antibiotics are also responsible for facilitating the emergence and spread of antibiotic-resistant microorganisms Thus, understanding farmers’ level of knowledge and practices towards the prudent use of antibiotics cannot be more emphasized than now. Nigeria being the most populated country in West Africa has to take the lead in tackling AMR using the ‘One Health’ approach, which acknowledges the links; humans, animals and the environment, as the cornerstone of its plan [11].

The objectives of this study are to assess the knowledge and practices among cattle handlers in Kwara State, Nigeria on antibiotic residues in meat and milk and to determine the association between knowledge and practice towards antibiotic residues. This study also explored a foundation on which to identify future opportunities for further research and initiatives relating to antimicrobial stewardship in Nigeria.

## Materials and methods

### Study design and location

**Fig 1:**
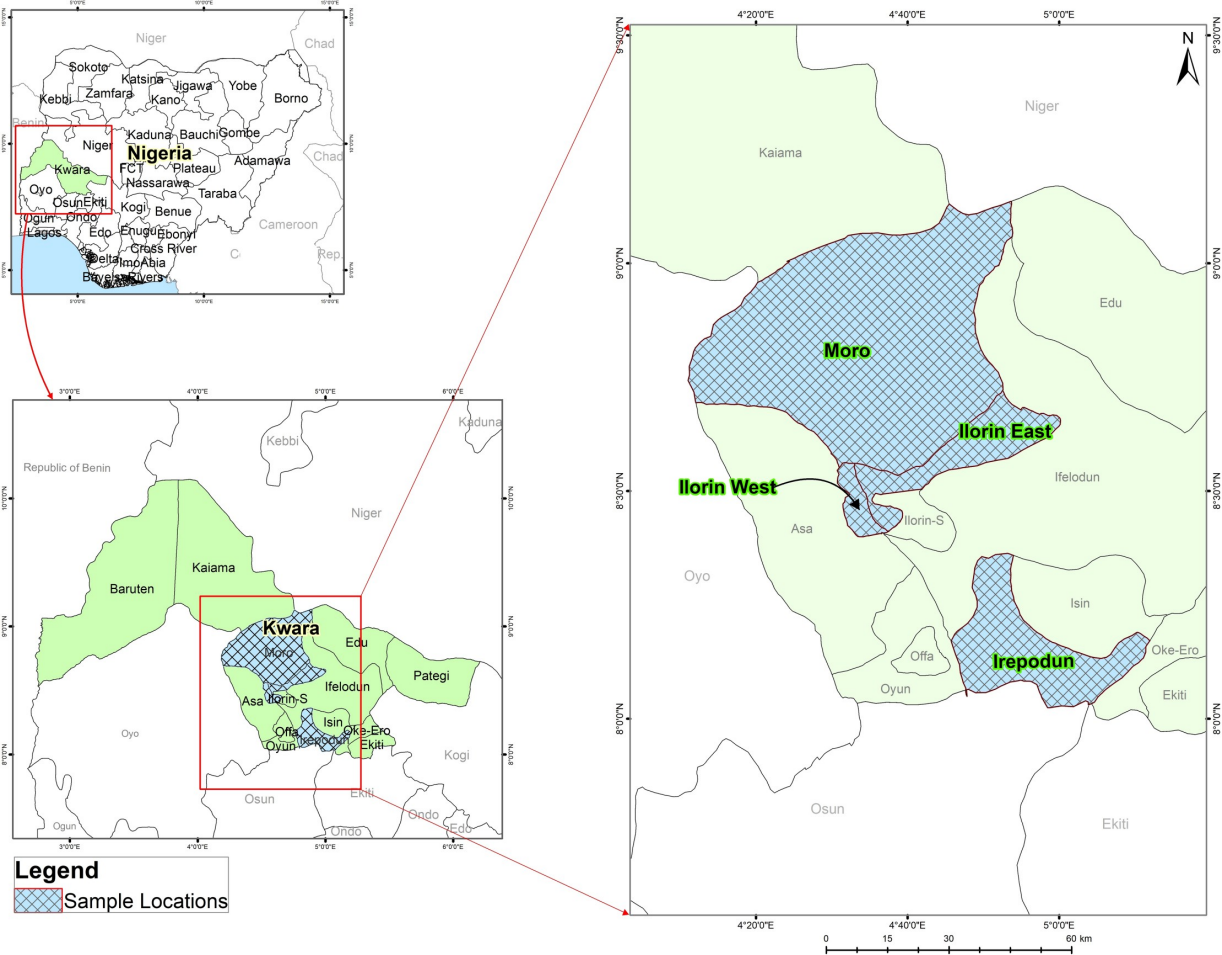
Spatial distribution of study areas in Kwara State, Nigeria.

A web-based cross sectional online survey was performed to determine the knowledge and practices of cattle handlers, regarding antibiotic use and residues in meat and milk. The study area is Kwara State in Western Nigeria, Fig 1. The State is located between Latitude 8.9848 N and Longitude 4.5624 E and in the North Central geopolitical zone, commonly referred to as the Middle Belt. Four Local Government Areas (LGAs) were randomly selected out of the 16 LGAs in the State. These states included Ilorin East, Ilorin West, Moro and Irepodun. The study population consisted of abattoir workers (especially those working at the ante-mortem inspection section of the abattoir), cattle sellers, pastoralists, cattle rearers/herders. This population was selected because they are the points of contact with the animals before antibiotics are administered in case of any disease or infection in the animals. The exclusion criteria included (i) abattoir workers whose job is to sell meat and do not possess cattle or influence the slaughtering of cattle, (ii) meat vendors whose job is to sell meat, (iii) milk and milk products vendors whose job is to sell milk and milk products to the consumers.

**Fig 2:**
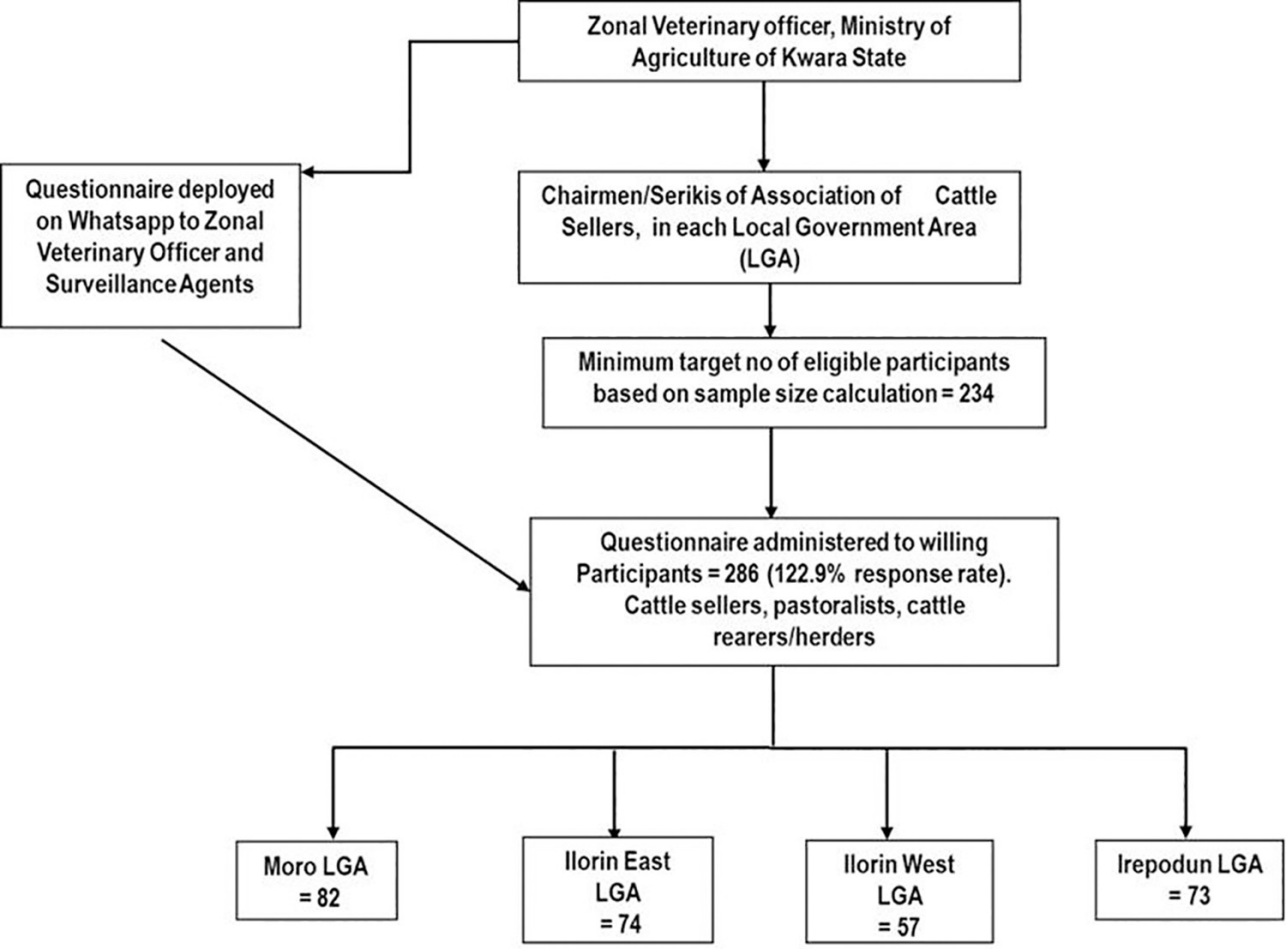
Recruitment flowchart for study participants.

Sample size determination, questionnaire and data collection. Sample size was calculated using the formula: *n* = Z^2^ *p* (1- *p*)/d^2^ Where; *n* is the sample size; z = confidence level (95% = 1.96); *p* = prevalence (15.4%) [12]; d = precision (Significance level) at 5%. The total sample size estimated was *n* = 211.Considering the non-response rate, 10% of the sample size was added to give a total of 234 participants. The non response rate was determined using the formula: *n*/1-f, (where f is non response rate = 10%). Prior to the commencement of the online survey, the Zonal Veterinary officer of Kwara State Ministry of Agriculture was contacted, who further recruited surveillance agents for each local government to be sampled. Through the help of the Zonal Veterinary officer and the surveillance agents, the chairmen/Serikis of Cattle Seller Association of each Local government area were also contacted and detailed information about the study aim and focus were discussed with them. Following their consent, the questionnaire was deployed on Whatsapp for the Zonal Veterinary officer, who later redeployed to other surveillance agents in each local government area and the survey was done using chain-referral sampling methodology. Fig 2 provides a flowchart for recruitment of participants.

The semi–structured interviewer-administered online questionnaire was designed in English Language on KoBo Toolbox software (KoBo Inc., Cambridge, MA 02138). The questionnaire consisted of three sections, which gathered information on the participants’ Socio-demographic characteristics, Knowledge and Practices regarding antibiotic residues in meat and milk. The section A) consisted of six (6) questions which assessed the socio-demographic profiles of the participants such as age, gender, years of experience, educational status, local government area and primary occupation. Section B comprised of a nine (9) item questions, which gathered on the knowledge of the participants towards antibiotic residues in meat and milk and these included questions such as have you heard of antibiotic residues before? Can a man consume antibiotics as a result of eating meat or drinking milk? Can antibiotic in meat and milk affect the consumer negatively? How can we avoid antibiotic in meat and milk? What is withdrawal period? Each correct answer weighs 1 point and incorrect or not sure answers weigh 0 point.

The last section consisted of an eleven (11) item questions assessed the practice levels of the participants regarding antibiotics residues and the questions included the following: do you ask when last the animal you are about to milk or slaughter? When antibiotics spill on the floor, do you clean it immediately? Has a Veterinary doctor ever stopped you from slaughtering or milking because you just finished giving antibiotics to the animal and what was your reaction? Have you ever used antibiotics without a Veterinary doctor’s prescription? How often do you give antibiotics to your animal? If a Veterinary doctor gave an antibiotic and it worked, do you use it again when animal is showing similar signs. Each correct answer weighs 1 point and incorrect or not sure answers weigh 0 point.

The questionnaire was pretested among 16 randomly selected cattle handlers in various locations in Ilorin, different from the study areas. The questionnaire was finalized after modifications based on the result of pretest. The responses from the pretest were not included in the final data analyzed. The online survey was carried out between 2 November 2019 and 9 December 2019. Detailed information on the questionnaire is presented in S1 File.

### Ethical consideration

Ethical approval for this study was obtained from The Ministry of Agriculture and Natural Resources, Ilorin, Kwara State, with reference number VKW-714/1/86. Informed verbal consent was obtained from participants. Participation was voluntary based on individual’s availability and willingness to be part of the study. All participants were notified of their right to discontinue at any stage of the survey [13].

### Statistical analysis

The data collected were exported from Kobo Toolbox application (Cambridge, MA) into Microsoft Excel® 2016 spreadsheet (Microsoft Corporation, Redmond, WA, USA) and variables were analyzed using Epi info version 7.1.3.10. Descriptive statistics such as frequencies and proportions and percentages were calculated. Bi-variate analysis was done by calculating the odds ratios and the level of statistical significance determined using Chi Square test at 95% Confidence Interval. To assess the knowledge and practice levels of cattle handlers regarding antibiotic residues, a numeric scoring system was used. The outcome variables were computed as binary responses such that ‘Yes’ and correct responses were scored as ‘1’ while ‘No’ and incorrect responses were scored as ‘0’. The grading system for knowledge ranged from 0 to 11, while that of practice ranged from 0 to 10 All scores were summed up and cut off points were set as follows: respondents scoring “<50%” or “>50%” were regarded as having “poor” or “good” knowledge and practice levels respectively [25]. Based on the cut off points, the cumulative grades were further categorized as ‘poor’ and ‘satisfactory’ to know the participants’ knowledge and practice levels regarding antibiotic residues in meat and milk in Kwara State.

Significant variables were further subjected to multivariate analysis using Epi info version 7.1.3.10 to determine potential factors influencing knowledge and practice levels among cattle handlers regarding antibiotic residues in meat and milk. Odds ratios (OR) were computed to determine of associations between variables at 95% Confidence Intervals (CIs).

## Results

### Demographic information of respondents

Out of the 286 participants interviewed, most were male 249 (87.1%) and 237 (82.9%) had working experience of 11 years and above. A greater proportion of the participants 206 (72%) were below the age of 40 while 147(51.4%) had secondary education. Table 1.

**Table 1:** Socio-demographic characteristics of respondents.

Variables	Proportion (%)	95% CI
	*n* = 286	
Gender		
Male	249 (87.1)	82.6–90.7
Female	37 (12.9)	9.3–17.4
Age		
< 40	206 (72.0)	66.4–77.2
≥ 40	80 (28.0)	22.9–33.6
Educational Status		
Tertiary	32 (11.2)	7.8–15.4
Secondary	147 (51.4)	45.4–57.3
Primary	68 (23.8)	19.0–29.1
Quranic	31 (10.8)	7.5–15.0
Not Educated	8 (2.8)	1.2–5.4
Business Duration		
< 11 years	49 (17.1)	13.0–22.0
≥ 11 years	237 (82.9)	78.0–87.1
Local Government Authority		
Ilorin East	74 (25.9)	20.9–31.4
Ilorin West	57 (19.9)	15.5–25.04
Irepodun	73 (25.5)	20.6–31.0
Moro	82 (28.7)	23.5–34.3
Primary Occupation		
Abattoir worker	94 (32.9)	27.5–38.6
Cattle Trader	123 (43.0)	37.2–49.0
Pastoralist	69 (24.1)	19.3–29.6

*n*: number of responses; CI: Confidence Interval

### Respondents’ knowledge on antibiotic residues

The knowledge of cattle handlers on antibiotic residues in meat and milk in Kwara State is described in detail in Table 2. A greater percentage 165 (52.7%) had no knowledge about antibiotic residues. Out of the 121 (42.3%) that have heard about ABR, 111 (91.7%) indicated that ABR could be caused by giving antibiotics to animals routinely and that slaughtering or milking animals immediately after administering drugs. Moreover, some respondents 129 (45.1) did not know if antimicrobial residues could accumulate in man from the consumption of meat and milk containing the residues. Likewise, 156 (54.6%) did not understand the term withdrawal period while 117 (40.9%) indicated withdrawal period as either the time it takes to stop administering antibiotics again or the time that should be allowed after giving the antibiotics before slaughtering or milking the animal. Only 113 (39.5%) of the respondents knew that consumption of meat and milk containing antimicrobial residues can affect the consumer health negatively and 28 (9.8%) indicated that we can avoid antibiotics in meat and milk by not slaughtering or milking an animal that is still on treatment.

**Table 2:** Knowledge of participants on antibiotic residues in meat and milk in Kwara State.

Variables	Proportion (%)	95% CI
Have you heard of antibiotic residues in meat or in milk before?		
Yes	121 (42.3)	36.5–48.3
No	165 (52.7)	51.7–63.5
If yes, what causes it? (n = 121)		
Giving antibiotics to animals routinely	4 (3.3)	0.9–8.3
Slaughtering/milking animal immediately after medication	3 (2.5)	0.5–7.1
All of the above	111 (91.7)	85.3–96.0
None of the above	1 (0.8)	0.02–4.5
I don’t know	2 (1.7)	0.2–5.8
Can man consume antibiotics as a result of eating meat/drinking milk?		
	
Yes	107 (37.4)	31.8–43.3
No	50 (17.5)	13.3–22.4
I don’t know	129 (45.1)	39.2–51.1
What is withdrawal period?		
The time it takes to stop giving antibiotics again	3 (1.1)	0.2–3.0
The time that should be allowed after giving the antibiotics and slaughtering/milking the animal	7 (2.5)	1.0–5.0
The time to withdraw from giving the antibiotics	3 (1.1)	0.2–3.0
All of the above	117 (40.9)	35.2–46.9
I don’t know	156 (54.6)	48.6–60.4
Can consuming antibiotics in the meat/milk of animals affect the consumer negatively?		
	
Yes	113 (39.5)	33.8–45.4
No	53 (18.5)	14.2–23.5
Not sure	120 (43.0)	36.2–47.9
How can we avoid antibiotic residue in meat/milk?		
By giving smaller dose of drugs than the doctor advised	1 (0.4)	0.01–1.9
By giving the same dose of antibiotics but smaller number of days than the doctor said	7 (2.5)	1.0–5.0
By not slaughtering/milking animal that is still taking drug	28 (9.8)	6.6–13.8
All of the above	140 (49.0)	43,0–54.9
I don’t know	110 (38.5)	32.8–44.4
The more antibiotics I give to my animal, the healthier the animal becomes		
	
Yes	261 (91.3)	87.4–94.3
No	25 (8.7)	5.7–12.6
The more antibiotics I give to my animal, the bigger the animal becomes		
	
Yes	118 (41.3)	35.5–47.2
No	168 (58.7)	52.8–64.5
Even if man consume antibiotics in meat/milk, it will only make him healthier		
	
Yes	68 (23.8)	19.0–29.1
No	218 (76.2)	70.9–81.0
I should wait for some time after administering antibiotics to my animal before slaughtering/milking		
	
Yes	210 (73.4)	67.9–78.5
No	76 (26.6)	21.6–32.1
Do you think if antibiotics are given too much to animals, it can cause any serious problem to man through meat and milk?		
	
Yes	106 (37.1)	31.5–43.0
No	37 (12.9)	9.1–17.4
Not sure	143 (50.0)	44.1–56.0

### Respondents’ practices on antibiotic residues

Antibiotics were self-administered to animals and without Veterinary’s prescription by 130 (45.5%) participants. Table 3. Majority of the cattle handlers 280 (98%) used antibiotics when animals are sick. When Veterinarians gave animals antibiotics that worked, 136 (47.6%) indicated they re-used the same antibiotics again when animals showed similar signs. Only 14 (4.9%) of the participants recorded when last the animal was given antibiotics before slaughtering or milking. Meanwhile, 59 (20.6%) reported they had been stopped by a Veterinarian from slaughtering or milking due to non-compliance with antibiotic withdrawal period. Out of the 59, 3 (5.1%) opposed the Vet. while 52 (88.1%) complied.

**Table 3:** Practices of participants regarding antibiotic residues in meat and milk in Kwara State.

Variables	Proportion (%)	95% CI
Do you ask when last the animal you are about to slaughter/milk, was given drug?		
	
Yes	26 (9.1)	6.02–13.04
No	206 (72.0)	66.4–77.2
Once in a while	54 (18.9)	14.5–24.0
When antibiotics spill on the floor, do you clean it immediately?		
Yes	204 (71.3)	65.7–76.5
No	45 (15.7)	11.7–20.4
Once in a while	37 (12.9)	9.3–17.4
If yes, why? (n = 203)		
It will dirty/stain the floor	100 (49.3)	42.2–56.4
It can be slippery	2 (1.0)	0.1–3.5
Healthy animals can lick it	99 (48.8)	41.7–55.9
Other reasons	2 (1.0)	0.1–3.5
Has a veterinary doctor ever stopped you from slaughtering/milking your animal because you just finished giving drug?		
	
	
Yes	59 (20.6)	16.1–25.8
No	183 (64.0)	58.1–69.6
I can’t remember	44 (15.4)	11.4–20.1
If yes, what was your reaction		
I opposed him	3 (5.1)	1.06–14.2
I obeyed him	52 (88.1)	77.1–95.1
I can’t remember my reaction	4 (6.8)	1.9–16.5
Have you ever used antibiotics without a Veterinary doctor’s prescription?		
	
Yes	130 (45.5)	39.6–51.4
No	128 (44.8)	38.9–50.7
I can’t remember	28 (9.8)	6.6–13.8
If yes, how oftenn = 127		
Once in a while	65 (51.2)	42.2–60.2
I often do that, I know my animals’ need	56 (44.1)	35.3–53.2
I always do that, I already know the drugs the animals use	6 (4.7)	1.8–10.0
How often do you give antibiotics to your animals?		
Every week	1 (0.4)	0.01–1.9
Every month	3 (1.1)	0.2–3.0
I only use antibiotics when the animal is sick	280 (98.0)	95.5–99.0
I don’t use antibiotics at all	2 (0.7)	0.1–2.5
If a Veterinarian gives you a drug and it works, do you use it again when the animal is showing similar signs		
	
Yes	136 (47.6)	41.6–53.5
No	104 (36.3)	30.8–42.2
Sometimes	46 (16.1)	12.0–20.9
Before your animal is slaughtered / milked, do you check for when last it was given antibiotics?		
	
Yes	14 (4.9)	2.7–8.1
No	210 (73.4)	67.9–78.5
Once in a while	62 (21.7)	17.0–26.9

### Multivariate analysis for the association between sociodemographic profiles of participants and their knowledge level on antibiotic residues

Table 4 summarizes the multivariate analysis of the association between socio-demographic characteristics and knowledge of participants on antimicrobial residues. The age, sex and business duration of participants were significantly associated with the cattle handlers’ knowledge of antimicrobial residues. The effect of education on knowledge was modified by age. Among the educated respondents, those who were ≤40 years old were 4 times the odds of having poor knowledge compared to those >40 years in age (Stratum Specific OR = 3.65; CI = 1.2, 11.1; p = 0.026).

**Table 4:** Multivariate analysis of the Association between socio-demographic characteristics and knowledge of respondents on antibiotic residues.

Variable	Category	Knowledge (%)		Stratum specific OR (95% CI)	Crude OR (95% CI)	P-Value X^2^
Please remove		Poor	Satisfactory			
	Covariate	Education Status				
Age	≤40	82	56	3.65 (1.2, 11.1)	1.19 (0.73, 1.95)	0.026
	> 40	61	64	0.87 (0.4, 1.7)		
	Covariate	Education Status				
LGAs	Ilorin East	40	59	0.46 (0.12, 1.78)	1.19(0.12, 1.30)	…
	Ilorin west	70	59	1.6 (0.50, 5.07)	….	…
	Irepodun	63	100	… …	….	…
	Moro	69	75	1.02 (0.09, 11.76)	….	…
Gender	Female	9	20	.	0.39 (0.19, 0.78)	0.006
	Male	91	80	… …		
Years in Business	< 11	6	36	… …	0.11 (0.06, 0.24)	0.001
	≥ 11	94	64	.		

OR = Odds Ratio; Confidence Interval at 95%; X^2^ = Chi square

Female respondents were less likely to have poor knowledge than their male counterparts, p<0.05 (OR = 0.39; CI = 0.19, 0.78; p = 0.006). Likewise, years of business duration of respondents had a strong interaction with knowledge of ABR. The odds of having satisfactory knowledge reduces among participants who had spent 11 years and above in business (OR = 0.11; CI = 0.06, 0.024, p = 0.001) compared to participants who had less than 11 years’ business experience.

However, the general knowledge level was estimated to be poor. Only 36.7% had satisfactory knowledge.

### Multivariate Analysis for the association between sociodemographic profiles of participants and their practice level on antibiotic residues

Furthermore, the practice of participants regarding antimicrobial residues was generally poor as only 31.5% had satisfactory practice scores. Table 5. Knowledge levels of participants were significantly associated with their practice levels. (OR = 2.43; CI = 1.45. 4.06; p = 0.001).

**Table 5:** Association between demographic characteristics and practice of respondents on antibiotic residues.

Variable	Category	Practice (%)		OR	95% CI	P value
		Poor	Satisfactory			
Age	> 40	70.4	29.6	1.35	0.78, 2.33	0.278
	≤ 40	63.8	36.3			
Gender	Female	66.7	33.3	0.47	0.19, 1.11	0.078
	Male	81.1	18.9			
^E^Year of Business	< 11	79	91	0.39 1.40	0.07, 2.14 0.63, 3.11	0.180
	≥ 11	88	84			
Knowledge	Poor	75.7	24.3	2.43	1.45, 4.06	0.001
	Satisfactory	56.2	43.8			

E = stratified by Education status

## Discussion

The issue of antibiotic residues in meat and milk is a serious problem that is not effectively addressed in low and middle income countries including Nigeria. The safety of foods of animal sources regarding drug residues receives suboptimal attention in the country and concerns on the public health impact such as antibiotic resistance of bacteria strains in humans and animals are growing. `

This study revealed that knowledge and practice regarding antibiotic residues were generally low among cattle handlers in Kwara State. This report is similar to studies conducted by [14–16] on livestock farmers in Sudan, Ghana and Cambodia respectively. A lower percentage of the participants understood that the presence of antibiotics in meat and milk was as a result of antibiotic residues in animal meat and milk and this corroborates the report by [17] who showed that only 35% of the respondents were aware. More than half of the participants had poor knowledge of the concept of withdrawal period and two-thirds affirmed waiting for some time after administering antibiotics before slaughtering or milking and this is in agreement with the report of [18] in whose report some risk factors for food contamination such as non adherence of withdrawal requirement of drugs, were related to lack of knowledge about withdrawal requirement of drugs. Withdrawal periods for meat and milk are listed on the data sheet accompanying the drug and farmers are supposed to strictly adhere. The poor knowledge observed in this study suggests that collaborative efforts in improving sensitization or educational programmes on antimicrobial stewardship are crucial. It might also be as a result of the educational status of the participants as most of them had secondary school education.

Regarding practices, about half of the cattle handlers self-reported using antibiotics without veterinarian’s prescription. In the same vein, about half of the participants indicated self-prescribing and repeating antibiotics treatment previously administered by veterinarians for animals when presenting similar signs. This is in line with the study conducted by [19] among Pastoralists in North-Central Nigeria who practiced self prescription and administration without professionals’ consultations. In Nigeria, a large amount of antimicrobials goes into use without Veterinarians’ prescriptions particularly as antibiotics are available over-the-counter (OTC) [20–23]. Sharma et al. [24] also described the use of old prescriptions as observed in this study. Farmers or cattle handlers result to self-medication due to lack of adequate and high cost of veterinary services as reported by [25]. This widespread and unrestricted usage of different antibiotics in food animals without adequate diagnosis, prescription and supervision of veterinarians has contributed greatly to the deposition of the residues in animal products [26]. The prevention levels of antibiotics beyond Maximum Residual Limits (MRLs) require combined and coordinated effort among government agencies, Veterinarians and livestock producers. As it obtains in High Income Countries (HICs), the avoidance of meat and milk residues in the livestock industry should take an on-farm-team-effort that begins with the VCPR-the Veterinary-Client-Patient-Relationship [27]. The cattle farm owner/manager/herdsman must work with the farm veterinarian to develop treatment protocols that address the judicious use of antibiotics. Once a decision is made to use antibiotics, protocols must then be put in place to guide employees on the safe way to handle this animal to prevent inadvertent meat and milk residues from occurring. Essentially, treated animals should be identified and antibiotic use must be recorded to prevent residues [27]. Therefore, to combat antibiotic residues in meat and milk and antimicrobial resistance, proper legislation must be set up to reinforce efforts to mitigate the overuse of drugs in livestock production.

In this study, participants’ demography such as age (*p* = 0.0026), gender (*p* = 006), years of business experience (*p* = 0.001), were significantly associated with knowledge on antibiotic residues. The effect of educational status on the knowledge of antibiotic residue was modified by age and this generated some divergent results. Participants who were below the age of 40 years and educated demonstrated poor knowledge on ABR, which means that level of education, did not necessarily translate into satisfactory knowledge. On the contrary, education was weakly protective against poor knowledge of ABR when a person is greater than 40 years of age. Participants above the age of 40 years and educated were more likely to have good knowledge of ABR than those below the age of 40 though educated (Stratum Specific OR = 3.65; CI = 1.2, 11.1; *p* = 0.026). Education was expected to have a positive association with knowledge as reported by some authors [14, 28, 29]. This poor knowledge observed among the young educated cattle handlers could have resulted from lack of awareness of the issues of antimicrobial residues, carefree attitude as well as lack of experience compared to the older cattle handlers who might have gained experience over the years. In addition, female respondents were less likely to have poor knowledge of antibiotic residues than their male counterparts and this is in agreement with the reports of [25] in which female participants were more likely to have better knowledge than their male counterparts (OR = 0.39; CI = 0.19, 0.78; *p* = 0.006). On the contrary, a previous report by [29] documented male participants were more likely to have better knowledge than female counterparts. There should be more awareness of antibiotic residues and resistance among the male folks in the livestock industry and also among the younger and educated cattle handlers.

Furthermore, the years of business duration was found to be positively associated with knowledge. Participants with 11 years and above in business were more likely to have satisfactory knowledge than those who had less than 11 years of experience. This finding was contrary to the finding of [30]. The present study also revealed no significant association between the demo-graphs of participants and their practice regarding antibiotic residues in meat and milk which is similar to the study conducted by [30]. Participants’ education, working experience, knowledge and attitude levels did not have any impact on practice levels on meat safety and sanitation [30].

Finally, knowledge levels of cattle handlers were significantly associated (*p*<0.05) with their practice. Several authors have reported significant association between knowledge and practice.

There were some limitations encountered in this study. A non-probabilistic convenience sampling method was used among cattle handlers in Kwara State, which was limited by location; therefore the findings may not be generalizable for other locations in the country. Also, it was specie-bound, mainly for bovine specie and the findings may not be conclusive for other specie handlers in the livestock industry.

## Conclusion

The present survey contributes to the better understanding of the current status of cattle handlers’ levels of knowledge and practice regarding antibiotic residues in Kwara State. The study outcomes provide basis for further research to detect and quantify the levels of antibiotic residues in edible meat and milk in Kwara State; with a view to knowing future opportunities for further research and initiatives. Deliberate efforts to prevent antibiotic residues in meat and milk should entail creation of awareness through periodic educational trainings, compliance with withdrawal period, effective surveillance systems and monitoring to control the use of veterinary drugs in Kwara State and the role of government is empirical to solving the problem of antimicrobial resistance in Nigeria.

## Supporting information

S1 File Questionnaire on the knowledge and practice of cattle handlers on antibiotic residues in meat and milk in Kwara State, Northcentral, Nigeria. (DOC)

## Abbreviations

ABR: Antibiotic Residues
AMR: Antimicrobial Resistance
CAADP: Comprehensive Africa Agriculture Development Programme
CI: Confidence Interval
FAO: Food and Agriculture Organization
FMAE&H: Federal Ministries of Agriculture, Environment and Health
FMARD: Federal Ministry of Agriculture and Rural Development
GDP: Gross Domestic Product
HICs: High Income Countries
HIV: Human Immunodeficiency Virus
LGAs: Local Government Areas
LMICs: Low and Middle Income Countries
OR: Odds Ratio
OTC: Over-the-counter
TB: Tuberculosis
VCPR: Veterinarian-Client-Patient-Relationship
WHO: World Health Organization
X^2^: Chi Square
