# Supplementary material for: Knowledge and practice of cattle handlers on antibiotic residues in meat and milk in Kwara State, Northcentral Nigeria
Source: PLoS One. 2021 Oct 14;16(10):e0257249. doi: 10.1371/journal.pone.0257249 (PMC8516246; doi:10.1371/journal.pone.0257249)
Supplement: S1 File — (DOC) [file pone.0257249.s001.doc]

**KNOWLEDGE, ATTITUDE AND PRACTICE OF ABATTOIR WORKERS/CATTLE OWNERS ON ANTIBIOTIC RESIDUE IN MEAT AND MILK, KWARA STATE, NIGERIA.**

**QUESTIONNAIRE**

Date ……/……/……….… Questionnaire No………….

Dear Respondent,

I am Dr. Mary Olasoju, student of University of Ibadan. I wish to seek your permission to administer this questionnaire, designed to assist us provide information on knowledge and practices of cattle handlers on antibiotic residues in milk and meat and their public health importance. We are hopeful that your sincere response to these questions will enable us achieve this objective. Your name and identity will not be required and all information provided would be used confidentially and solely for the purpose of this study. Your participation is voluntary.

The period for this interview will last for about 20 minutes; you are free to stop the interview at any time.

Thank you for your kind assistance.

**SECTION A:SOCIO-DEMOGRAPHIC CHARACTERISTICS**

1. Location………………………………………….
2. LGA……………………………………………………….
3. Gender: Male Female
4. Age (in years)
5. Highest educational status attained

a. Quranic b. Primary c. Secondary

d. Tertiary e. Informal

7. Main Occupation

a. Abattoir worker b. Pastoralist

c. Cattle Trader d. Others (specify)………………………………………….

1. How long have you been in the business (in years)? …………………………

**KNOWLEDGE**

1. Have you heard of antibiotic residues in meat or in milk before?

Yes No

1. If yes, what causes it?

Answer…..

1. Can man consume antibiotics as a result of eating meat/drinking milk?

Yes No Not sure

1. What is withdrawal period?
2. The time it takes to withdraw from given antibiotics again
3. The time that should be allowed after given the antibiotics and slaughtering/milking the animal
4. The time to withdraw from given the antibiotics

1. All of the above
2. I don’t know
3. Can consuming antibiotics in the meat/milk of animals affect the consumer negatively?

Yes No Not sure

1. What can lead to antibiotic residue in meat/milk of animals?
2. Given antibiotics to animals routinely
3. Milking/Slaughtering the animal immediately after it has finished medication
4. All of the above
5. None of the above
6. I don’t know
7. How can we avoid antibiotic residue in meat/milk?
8. By given smaller dose of drugs than the doctor advised
9. By given the same dose of antibiotics but smaller number of days than the doctor said
10. By not slaughtering/milking animal that is still taking drug
11. All of the above
12. I don’t know
13. Which of the following statements/are is true?
14. The more antibiotics I give to my animal, the more healthier the animal becomes
15. The more antibiotics I give to my animal, the more bigger the animal becomes
16. Even if man consume antibiotics in meat, it will only make him healthier
17. I should wait for some time after administering antibiotics to my animal before milking/slaughtering
18. I don’t know
19. Do you think if antibiotics are given too much it can cause any serious problem to man?

Yes No Not sure

PRACTICE

| Variables | Choices |
| --- | --- |
| 11 .Do you ask when last the animal you are about to milk/slaughter was given drug? | Yes No |
| 1. When antibiotics spills on the floor, do you clean it immediately? | Yes No |
| 12b. If your answer to the above is yes, why? | 1. It will dirty/stain the floor 2. It can be slippery 3. Other reasons please state…… |
| 1. Has a veterinary doctor ever stopped you from slaughtering/milking your animal because you just finished giving drug? | Yes No |
| 13b. What was your reaction? | 1. I obeyed him 2. I opposed him 3. I can’t remember my reaction |
| 1. Have you ever used antibiotics without a Veterinary doctor’s instruction/prescription? | Yes No |
| 14b. If your answer to the above is yes, how often do you do this? | 1. Once in a while 2. I often do that (I know my animals’ need) 3. I always do that (I already know the drugs the animals use) |
| 1. How often do you use antibiotics? | 1. Every week 2. Every month 3. I only use antibiotics when the animal is sick 4. I don’t use antibiotics at all |
| 1. If a Veterinarian gives you a drug and it works, do you use it again when the animal is showing similar signs | 1. Yes 2. No 3. sometimes |
| 1. Before your animal is milked/slaughtered do you check for when last it was given antibiotics? | Yes No |
| 1. If yes, how often do you do this? | 1. Once in a while 2. Quite often 3. Always |
